# The Effect of Trans-Polyoctenamer on Thermoplastic Dynamic Vulcanizates from Devulcanized Ground Tire Rubber and Polypropylene

**DOI:** 10.1021/acsomega.6c03249

**Published:** 2026-06-15

**Authors:** Ákos Görbe, Gergő Zsolt Marton, Tamás Bárány

**Affiliations:** † Department of Polymer Engineering, Faculty of Mechanical Engineering, 61810 Budapest University of Technology and Economics, Műegyetem rkp. 3, Budapest H-1111, Hungary; ‡ MTA-BME Lendület Sustainable Polymers Research Group, Műegyetem rkp. 3, Budapest H-1111, Hungary

## Abstract

In this study, we investigated the effect of trans-polyoctenamer (TOR) on the properties of thermoplastic dynamic vulcanizates (TDVs) derived from devulcanized ground tire rubber (dGTR) and polypropylene (PP). Small amounts of TOR helped improve the interface between incompatible PP and dGTR through co-cross-linking of TOR and the rubber phase at the interphase and slightly increased the crystallinity of the thermoplastic phase. The improved interface manifested in the higher elongation-at-break and perforation energy and improved compression set. We also studied the damage behavior of the TDVs and found that the addition of TOR led to a more stable, gradual failure process, as evidenced by significantly flatter cumulative acoustic emission event curves.

## Graphical abstract

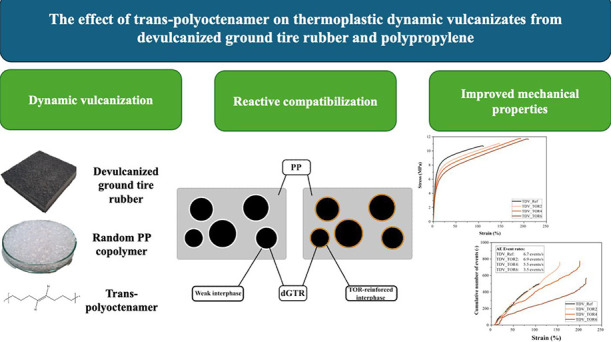

## 1 Introduction

The recycling of polymer waste remains one of the most important and extensively researched areas of polymer science. While thermoplastic materials can be reprocessed relatively easily through remelting and reshaping,^1,2^ rubber waste poses a significant challenge due to its cross-linked structure, which prevents reversible melting and conventional recycling methods.^3–5^ Among the various strategies developed to address this issue, the production and reuse of ground tire rubber (GTR) have emerged as being widely adopted and cost-effective approaches. GTR is commonly utilized as a filler in applications such as asphalt pavements, flooring, playground surfaces, and polymer composites.^6,7^

Incorporating GTR into thermoplastic polymers can offer advantages such as improved impact resistance and reduced material costs.^8,9^ However, the inherently poor compatibility between GTR and common thermoplastics (particularly polypropylene (PP)) limits its application at higher loading levels. To overcome this limitation, surface activation or structural modification of GTR is necessary.^10–12^ One of the most promising approaches is devulcanization, a process that selectively cleaves sulfur cross-links in vulcanized rubber via targeted energy input. This treatment yields devulcanized ground tire rubber (dGTR), a partially reclaimed material that regains flowability and reactivity.^13–15^

The incorporation of dGTR into thermoplastic matrices enables the fabrication of thermoplastic dynamic vulcanizates (TDVs): a class of thermoplastic elastomers characterized by finely dispersed rubber domains within a thermoplastic phase.^16–18^ These materials are produced via dynamic vulcanization, in which the rubber phase is vulcanized in situ during melt processing, typically in a twin-screw extruder.^19–21^ Among commercial TDVs, the PP/EPDM system is the most widely used. Replacing virgin rubber with dGTR in TDVs offers a promising route to upcycle end-of-life tires into value-added, recyclable materials, thereby supporting the transition toward a circular economy.^22,23^

Despite the improved processability of dGTR, good dispersion and interfacial adhesion between the rubber phase and the thermoplastic matrix remain key factors for high-performance TDVs. The inherent incompatibility between apolar thermoplastics such as PP and the more polar, heterogeneous surface of dGTR often results in poor phase adhesion, thereby reducing the mechanical performance. Therefore, compatibilization strategies are required to enhance the interfacial interactions between the two phases.^11,24–28^

One effective approach involves the use of functionalized polymers or oligomers that can interact with both the thermoplastic matrix and rubber filler. Among these, trans-polyoctenamer rubber (TOR) has gained an increasing amount of attention. TOR is a semicrystalline polyolefin rubber with a trans-configuration, known for its low molecular weight, high chain mobility, and chemical structure similar to that of natural rubber and diene-based rubbers. Owing to its unsaturated backbone, TOR can co-cross-link with the rubber phase, thereby improving interfacial adhesion between incompatible phases.^29,30^

In addition to its chemical compatibility with rubber, TOR also exhibits partial miscibility with polyolefins, making it a molecular bridge between the dGTR and the PP matrix.^31–34^ Its incorporation can lead to a more uniform dispersion of the rubber phase, reduced interfacial tension, and enhanced mechanical integrity of the final TDV. Moreover, the use of TOR may contribute to a more gradual evolution of damage and improved toughness by facilitating stress transfer across the phase boundary.^35^

Although TOR has demonstrated potential as a compatibilizer in various rubber systems, its effects on the performance and structure of TDVs based on PP and dGTR have not yet been fully understood. In this study, we address this gap by preparing a series of PP-based TDVs incorporating dGTR as the rubber phase and varying levels of TOR as a compatibilizer. The materials were produced via dynamic vulcanization using a twin-screw extruder and subsequently characterized by using a comprehensive set of techniques, including mechanical testing, morphological analysis, thermal analysis, and acoustic emission monitoring. Our goal was to evaluate the compatibilizing role of TOR, understand its impact on the microstructure and failure mechanisms, and identify an optimal formulation that balances mechanical performance and recyclability in dGTR-based TDVs.

## 2 Materials and Methods

### 2.1 Materials

We used PP R670 random polypropylene copolymer (extrusion grade, MFI = 2 g/10 min (2.16 kg, 230 °C) 2 g/10 min) as the matrix material, provided by MOL Petrochemicals Ltd. (Tiszaújváros, Hungary), based on previous results,^23^ where we explored the effect of viscosity ratio.

Vestenamer 8012 (Evonik Industries AG, Essen, Germany) trans-polyoctenamer was used as a compatibilizer for the TDV phases.

dGTR was provided by Tyromer Inc. (Waterloo, ON, Canada). GTR, made from truck tires with a particle size of less than 1 mm, was thermomechanically devulcanized in an extruder with the help of supercritical CO_2_.

The curing agents and additives in the rubber phase of the TDVs are given in Table 1.

**Table 1:** Materials Used for Curing the Rubber Phase of the TDV

**material**	**manufacturer**	**trademark**	**function**
ZnO	Werco Metal (Zlatna, Romania)	-	activator
stearic acid	Oleon (Ertvelde, Belgium)	Radiacid 0154	
CBS N-cyclohexyl-2-benzothiazolesulfenamide	Rhein Chemie (Mannheim, Germany	Rhenogran CBS-80	accelerator
sulfur	Ningbo Actmix Polymer (Ningbo, Zhejiang, China)	ACTMIX S-80	curing agent

### 2.2 Preparation of the TDVs

The rubber mixture for the TDVs was prepared in a Brabender Lab-Station internal mixer (Brabender GmbH and Co. KG (Duisburg, Germany)) equipped with a W 350 E chamber (free volume 370 cm^3^) and a roller-type rotor. The temperature was set to 50 °C, and the batches were mixed at 40 rpm for 4 min at a fill factor of 80%. Table 2 shows the recipe of the rubber phase in parts per hundred rubber (phr).

**Table 2:** Recipe for the Rubber Mixture

	**amount of ingredient** (phr)
dGTR	100
ZnO	5
stearic acid	2
CBS	1.5
sulfur	1.5

The compounds were prepared with a corotating twin-screw extruder (Labtech Engineering Co., Ltd., Samutprakarn, Thailand, L/D = 44, screw diameter = 26 mm) at 120 rpm with a temperature profile ranging from 170 to 180 °C. The compositions of the compounds are listed in Table 3, and all components were introduced to the extruder in the first zone.

**Table 3:** Formulation of the Compounds

compound	components
TDV_ref	40 wt % PP R670 + 60 wt % dGTR mixture
TDV_TOR2	38 wt % PP R670 + 2 wt % TOR + 60 wt % dGTR mixture
TDV_TOR4	36 wt % PP R670 + 4 wt % TOR + 60 wt % dGTR mixture
TDV_TOR6	34 wt % PP R670 + 6 wt % TOR + 60 wt % dGTR mixture

We prepared flat and dumbbell specimens of both the TDVs and the reference PPs by injection molding with an Arburg Allrounder Advance 270S 400–170 type (Arburg GmbH, Lossburg, Germany) machine according to Table 4.

**Table 4:** Parameters for Injection Molding

temperature profile (from nozzle) [°C]	190/190/185/180/175/45
dosage [cm^3^]	45
residual cooling time [s]	20
injection rate [cm^3^/s]	25
back-pressure [bar]	40
mold temperature [°C]	30

### 2.3 Characterization Methods

We studied the cross-linking of the rubber phase and TOR during dynamic vulcanization with a MonTech D-RPA 3000 (MonTech (Buchen, Germany)) rubber process analyzer. We characterized three specimens using an anisotherm test conducted between 170 and 180 °C at a heating rate of 10 °C/min to model the temperature profile during extrusion.

The cross-link density of the samples was determined with the equilibrium swelling test method. Three replicate samples were soaked in toluene for 72 h at room temperature, as recommended by ASTM D6814–02(2018). After that, the samples were removed from the solvent and dried with paper towels, and the swollen mass was measured. The samples were then dried at 80 °C for 12 h, and their mass was measured again.

We calculated the cross-link density using the Flory–Rehner equation (eq 1).

$$\nu_{e}=\frac{-[\ln(1-V_{r}]+V_{r}+\chi \times V_{r}^{2}]}{\left[V_{s}\cdot \left(V_{r}^{1/3}-\frac{V_{r}}{2}\right)\right]}$$ 1

where ν_e_ is the cross-link density (mol/cm^3^), *V*_s_ is the molar volume of the solvent (for toluene: 106.27 cm^3^/mol), χ is the Flory–Huggins interaction parameter (0.391), and *V*_r_ is the volume fraction of rubber in the swollen network. *V*_r_ can be calculated with the Ellis–Welding equation (eq 2).

$$V_{r}=\frac{\frac{m_{r}}{\rho_{r}}}{\frac{m_{r}}{\rho_{r}}+\frac{m_{s}}{\rho_{s}}}$$ 2

where *m*_r_ is the weight of the dry sample (g), *m*_s_ is the weight of the solvent absorbed by the sample (g), ρ_r_ is the density of the rubber sample (g/cm^3^), and ρ_s_ is the density of the solvent (for toluene: 0.867 g/cm^3^).

We also determined the swelling index (%) of the samples using eq 3.

$$\mathrm{Swelling}\;\mathrm{index}=\frac{m_{\mathrm{sr}}-m_{r}}{m_{r}}$$ 3

where *m*_sr_ is the weight of the swollen sample (g).

The density of the samples was determined according to ASTM D 297–93 (hydrostatic method) using a Sartorius Quintix 125D semimicro balance with a resolution of 0.01 mg, with three repeats. The test medium was distilled water at 20.8 °C and a density of 0.998 g/cm^3^.

The morphology of the samples was examined using a JEOL JSM 6380 LA (Jeol Ltd., Tokyo, Japan) scanning electron microscope (SEM) with an accelerating voltage of 10 kV, a spot size of 40 nm, and magnifications of 100 and 1000. We examined cross sections of failed tensile test specimens to assess the phase contacts.

The crystallinity of the TDVs was measured by differential scanning calorimetry (DSC) and X-ray diffraction (XRD). The measurement was conducted on a Q2000 DSC device (TA Instruments, New Castle, United States) in the heat–cool–heat mode over the temperature range 0–200 °C, with a heating and cooling rate of 10 °C/min under a nitrogen atmosphere at 50 mL/min. The crystalline melting temperature was determined as the peak of the second heating curve, and crystalline melting enthalpy was determined as the area under the melting peak with respect to the baseline. The global crystallinity of the samples was determined with eq 4, and the normalized crystallinity was determined according to eq 5:

$$X_{\mathrm{glob}}=\frac{\Delta H_{m}}{\Delta {H_{m}}^{0}}\times 100$$ 4

$$X_{\mathrm{norm}}=\frac{\Delta H_{m}}{\Delta {H_{m}}^{0}\times (1-\alpha )}\times 100$$ 5

where *X*_glob_ is global crystallinity (%), Δ*H*_m_ is the melting enthalpy of the sample (J/g), $\Delta {H_{m}}^{0}$ is the melting enthalpy of a perfectly crystalline PP (207 J/g^36^), and α is the ratio of fillers in the material (−).

XRD measurement was performed on a Bruker D6 Phaser XRD device with a Cu K_α_ radiation source (Bruker Corporation, Billerica, USA). The crystallite size was estimated with the use of the Scherrer equation (eq 6).

$$D=\frac{K\lambda }{\beta \cos\theta }$$ 6

where *D* is crystalline size (nm), K is the shape factor, λ is the wavelength of the Cu Kα radiation (0.154 nm), β is the full width at half-maximum (fwhm) of the diffraction peak, and θ is the Bragg angle.

The compression set of the TDVs was determined according to ASTM D 395 at a 25% deflection, at 70 °C, and for 24 h, with five repetitions. We used eq 7 to calculate the compression set:

$$\mathrm{CS}=\frac{H_{0}-H_{2}}{H_{0}-H_{1}}\times 100$$ 7

where CS is the compression set (%), *H*_0_ is the height of the specimen before compression (mm), *H*_1_ is the height of the specimen during compression (mm), and *H*_2_ is the height of the specimen after compression (mm).

The abrasion resistance of the TDVs was measured on a standard rolling abrasion meter (Microvision Engineering Pvt. Ltd. (Rai Sonepat, India) according to ISO 4649:2024 with five repetitions. Abrasion loss was calculated with eq 8:

$$\Delta m=1-\frac{m_{1}-m_{2}}{m_{1}}\times 100\%$$ 8

where Δ*m* is abrasion loss (%), *m*_1_ is the mass of the specimen before the test (mg), and *m*_2_ is the mass of the specimen after the test (mg).

The surface of the abraded samples was studied with a Keyence VHX-5000 (Keyence Corporation (Mechelen, Belgium)) light microscope with a magnification of 20x.

We performed tensile tests in accordance with ISO 527 on type 1A dumbbell specimens with five replicates. A Zwick Z005 (Zwick GmbH (Ulm, Germany)) universal testing machine equipped with a 5 kN load cell was used at a crosshead speed of 100 mm/min.

We used the acoustic emission (AE) technique to monitor the damage and failure processes in the tested specimens during tensile testing. The method is based on detecting the acoustic waves generated by the material’s damage and failure mechanisms. Furthermore, this method enables characterization of these mechanisms by analyzing signal properties.^37^

Amplitude is a key parameter representing the peak voltage of the AE signal and is typically measured in decibels (dB). The signal strength can be determined as the time integral of the absolute value of the voltage signal, while absolute energy is defined as the time integral of the square of the voltage signal. These parameters are frequently used for damage analysis and may provide detailed information about the severity of the damage. Counts, which means the number of threshold crossings of the acoustic wave signal, may indicate the complexity of the event. The signal duration is the time interval between the first and last threshold crossings; generally, a longer duration indicates more gradual damage. Rise time, defined as the time between the first threshold crossing and the peak value, is also an important parameter. A shorter rise time may indicate sudden damage events, whereas a longer rise time may suggest a more stable damage mechanism. In addition, a frequency analysis of the signal can provide further information about the nature of the damage and may even help distinguish between different types of damage.^38,39^

We recorded AE signals during tensile tests using the measurement setup in Figure 1, with a Mistras PCI-2 (MISTRAS Group, Princeton Junction, USA) AE system connected to an IL40S preamplifier (Physical Acoustic Corporation, Princeton Junction, USA) with a gain of 40 dB and a Micros30s (Physical Acoustic Corporation, Princeton Junction, USA) microphone (operating frequency range: 150–400 kHz). To ensure proper connection between the sensor and the specimen, we used Oxett silicon grease (T-Silox Ltd., Budapest, Hungary) as a coupling agent. We set a 30 dB threshold for the measurements to filter out ambient noise, based on previous experience.^26^ The AE test results were evaluated using Noesis 9.0 and MATLAB R2024b.

**Figure 1:**
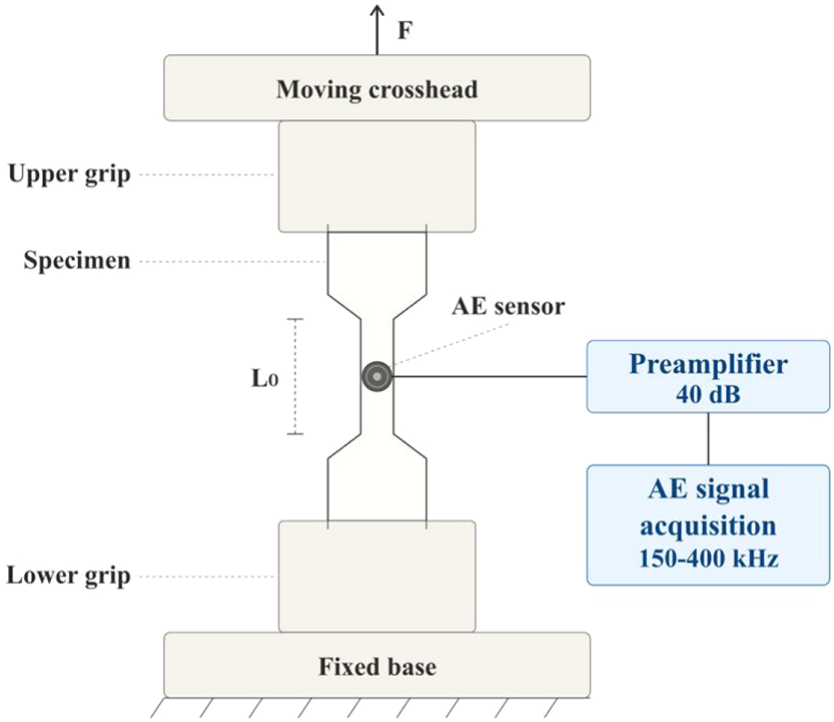
Measurement setup for detecting AE signals.

The injection-molded specimens were characterized with instrumented falling weight impact tests performed with a Ceast Fractovis 9350 falling weight impact testing machine (Instron, Torino, Italy) at room temperature, according to ISO 6603, on five repetitions. The total mass of the dart was 19.5 kg, the drop height was 1 m, the impact energy was 200.2 J, and the diameter of the dart was 20 mm, and a 40 mm diameter supporting ring was used to clamp the 80 × 80 mm square specimens.

## 3 Results and Discussion

### 3.1 Effects of TOR on the Cross-Linking of the Rubber Phase

The effects of TOR in the dGTR were initially evaluated through anisothermal vulcanization and cross-link density measurements (Figure 2 and Table 5). Anisothermal vulcanization is used to model cross-linking during dynamic vulcanization. The reference dGTR was characterized by the shortest vulcanization initiation time (*t*_10_) and optimal curing time (*t*_90_). The addition of TOR systematically delayed both the onset and the optimal vulcanization times, indicating its active participation in the cross-linking reactions. This shift suggests that TOR not only acts as a physical compatibilizer but also chemically integrates into the rubber network. Cross-link density measurements further confirmed these observations, revealing a consistent increase with elevated TOR content. TOR contains a high density of double bonds along its backbone, rendering it susceptible to sulfur-mediated cross-linking under the curing conditions employed. Co-vulcanization of TOR with the elastomeric component in PP/rubber systems using sulfur-based curing agents has been reported previously: Awang et al. demonstrated that TOR preferentially locates at the rubber–matrix interface and participates in the cross-linking reactions of the rubber phase.^32^ The increase in cross-link density and the change in curing kinetics observed in the present study are consistent with this established mechanism, and their concentration-dependent magnitudes are expected for a reactive component. The absence of TOR co-cross-linking without curing agents, as demonstrated by Zedler et al.,^34^ further supports the interpretation that the observed effects are chemically driven rather than purely physical.

**Figure 2:**
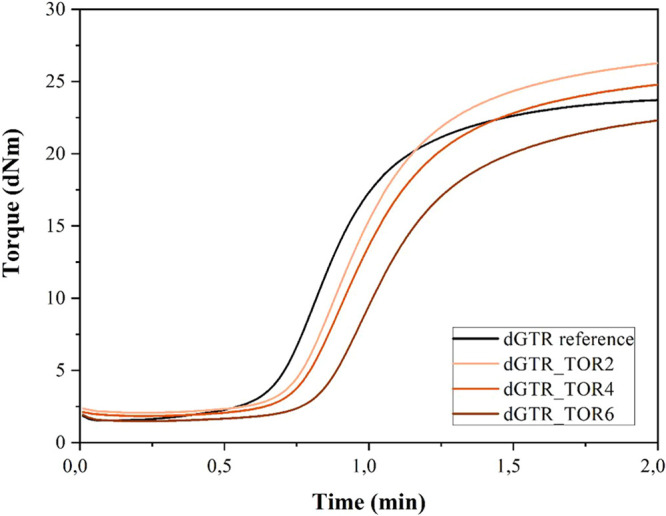
Vulcanization curves of the rubber phase with additional TOR.

**Table 5:** Vulcanization Results and Cross-Link Densities of the Rubber Specimens

specimen	*t*_10_ (min)	*t*_90_ (min)	cross-link density (×10^–4^mol/cm^3^)
dGTR reference	0.65	1.29	3.15 ± 0.23
dGTR_TOR2	0.74	1.42	3.60 ± 0.05
dGTR_TOR4	0.76	1.44	4.35 ± 0.78
dGTR_TOR6	0.83	1.51	5.36 ± 0.40

### 3.2 Crystallinity

DSC results (Figure 3 and Table 6) showed a slight increase in the melting temperature for the TOR-modified systems, indicating a modest improvement in the thermal stability of the crystalline phase. Crystallinity, normalized to the PP content, increased moderately with TOR, suggesting a slight improvement in the crystallization of the remaining PP fraction. However, the overall crystallinity of the TDV remained nearly constant, highlighting that the total crystalline fraction in the blend was unaffected by the TOR. Furthermore, the absence of any transitions attributed to the melting of the TOR indicates that the compatibilizer was successfully incorporated into the structure. The moderate increase in normalized PP crystallinity (counting only for the amount of PP in the TDV) with TOR content is attributed to the relief of crystallization constraints imposed by the rubber phase. As TOR improves phase compatibility, PP chains are liberated from interfacial disruption, enabling more complete crystallization of the thermoplastic fraction, consistent with the unchanged global crystallinity observed across all formulations.

**Figure 3:**
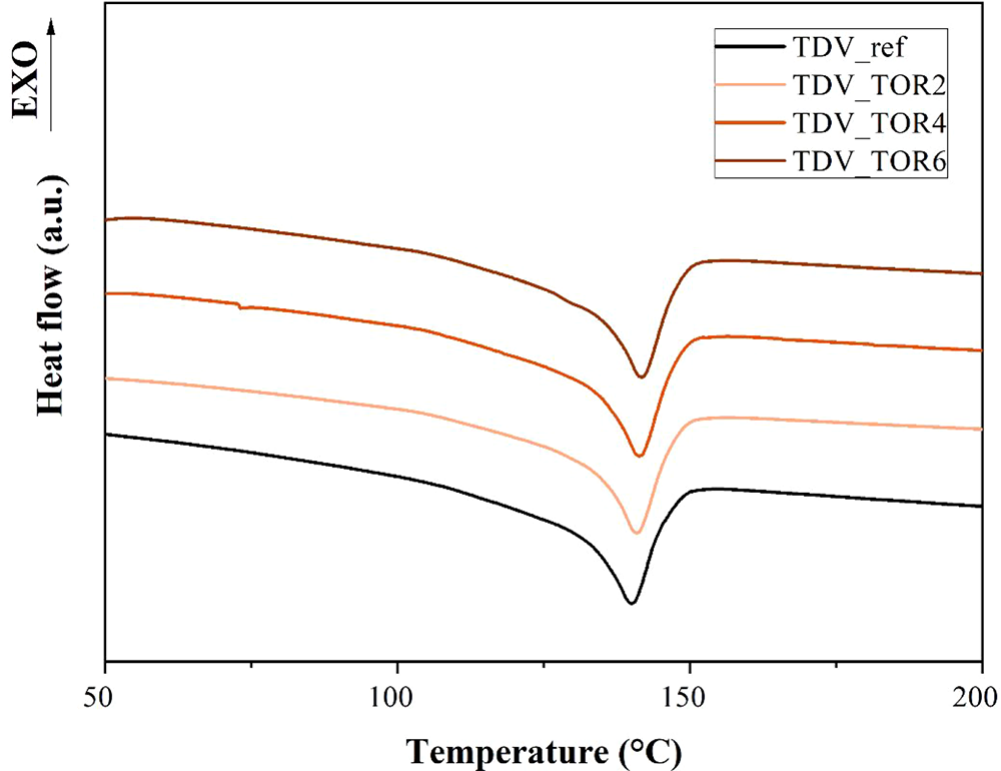
DSC curves of the TDVs.

**Table 6:** Results of DSC Measurements

specimen	melting temperature (°C)	normalized crystallinity (%)	global crystallinity (%)
TDV_ref	139.9	29.6	11.8
TDV_TOR2	140.9	30.9	11.8
TDV_TOR4	140.7	32.5	11.7
TDV_TOR6	140.7	33.7	11.5

The XRD analysis (Figure 4) revealed that the incorporation of TOR did not induce significant changes in the crystalline structure of the PP matrix. The main diffraction peaks remained at similar positions across all samples, indicating that the lattice parameters remained unchanged. The calculated crystallite sizes, derived from the Scherrer equation, were consistently 17–21 nm across all TOR contents. This suggests that TOR primarily influences the amorphous regions or the interfacial compatibility with the rubber domains, without altering the crystalline phase of the PP.

**Figure 4:**
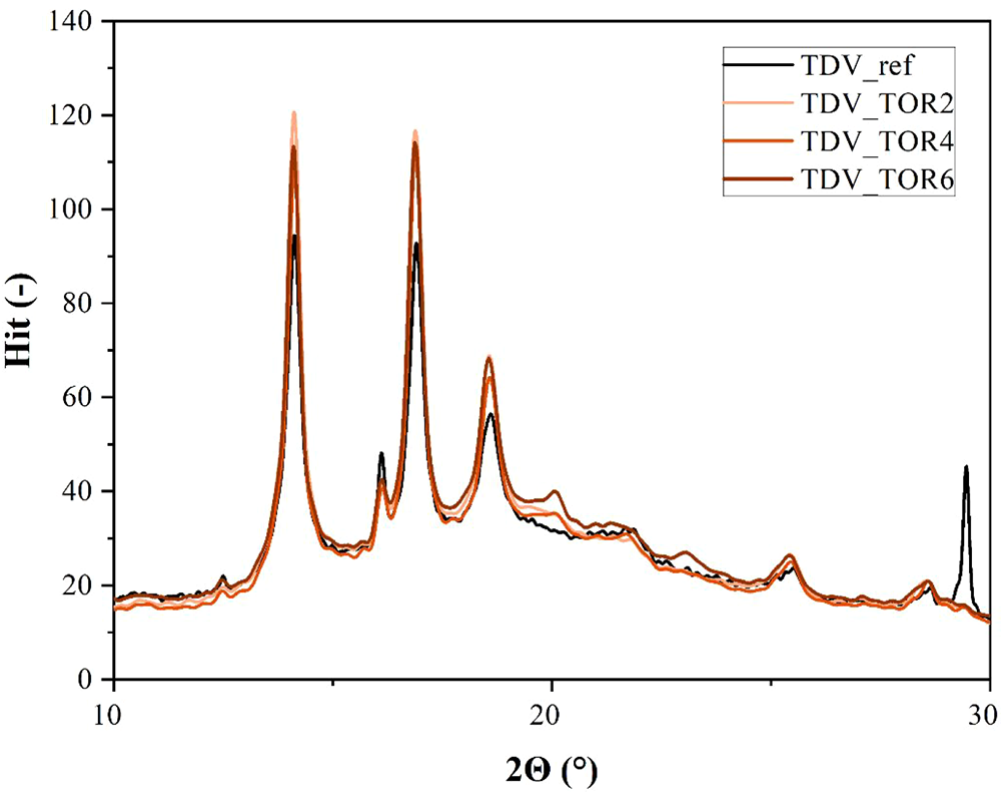
XRD spectra of the TDVs.

### 3.3 Compression Set and Abrasion Resistance

The compression set and abrasion resistance of TDVs (Table 7) are key indicators of their applicability. Compression set measurements further validated the beneficial effects of incorporating the TOR into TDVs. The reference material exhibited the highest compression set, indicating a limited elastic recovery and weaker network integrity. Increasing the TOR content caused a consistent decrease in the compression set.

**Table 7:** Compression Set and Abrasion Loss of the TDVs

specimen	compression set (%)	abrasion loss (%)
TDV_ref	30.8 ± 6.1	17.5 ± 0.5
TDV_TOR2	24.5 ± 4.6	15.3 ± 0.5
TDV_TOR4	21.5 ± 5.3	13.1 ± 0.5
TDV_TOR6	19.2 ± 1.6	12.1 ± 0.5

Similarly, abrasion loss decreased with increasing TOR content. The abraded surface (Figure 5) of the reference TDV exhibits a rough and heterogeneous morphology with numerous voids and traces of rubber-particle pullout, indicating dominant interfacial debonding during abrasion. In contrast, TOR-compatibilized TDVs show a more homogeneous wear surface with fewer pulled-out particles and more continuous abrasion tracks. This suggests improved interfacial adhesion between the PP matrix and the rubber phase, leading to a transition from interfacial failure-dominated wear to a more matrix-controlled and stable wear mechanism. These results demonstrate improved elasticity and enhanced network formation, attributable to effective cross-linking and improved interfacial interactions facilitated by TOR.

**Figure 5:**
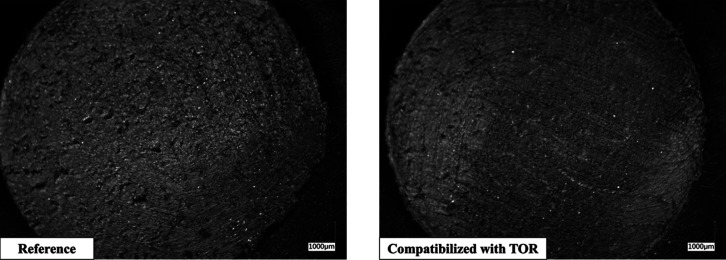
Surface of the abraded samples (without TOR and with TOR compatibilization).

Figure 6 shows a schematic illustration of the interfacial structure in the reference and the TOR-compatibilized TDVs. It should be noted that the PP and dGTR phases remain thermodynamically immiscible; the term ‘compatibility’ used here refers exclusively to improved interfacial interactions, including reduced interfacial tension and enhanced adhesion between the phases, rather than thermodynamic miscibility. In the reference system, poor compatibility between the PP matrix and the dGTR particles results in a weak interphase, promoting interfacial debonding and rubber-particle pullout during abrasion. This interfacial instability leads to localized material removal and a rough, heterogeneous worn surface, as shown in Figure 5. In contrast, the incorporation of TOR leads to the formation of a cross-linked, compliant interphase that improves interfacial cohesion and dissipates energy at the phase boundary, reducing stress concentration and stabilizing stress transfer between the matrix and the rubber phase. Consequently, the rubber particles remain better embedded in the PP matrix under mechanical loading, interfacial debonding becomes less dominant, and the governing deformation mechanism shifts toward a more-matrix-controlled response.

**Figure 6:**
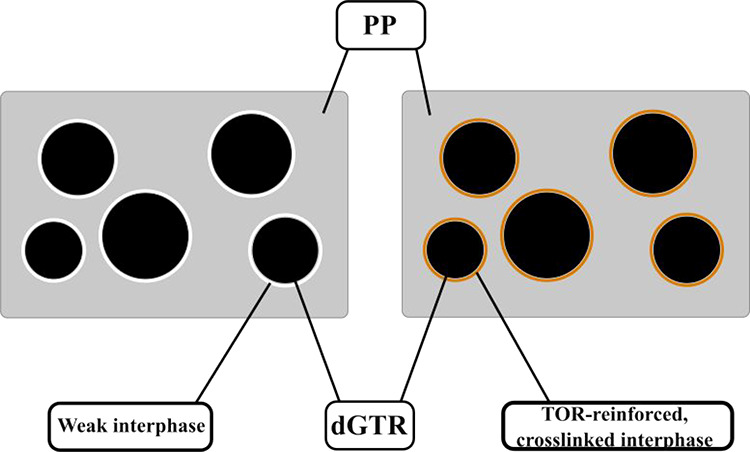
Schematic illustration of TOR compatibilization.

### 3.4 Morphology

Figure 7 presents SEM micrographs of the fracture surfaces of the TDVs (at 500× magnification). In the reference sample, the rubber particles exhibit sharp, well-defined contours and are clearly separated from the PP matrix. Distinct gaps at the phase boundary suggest poor interfacial adhesion, whereas the relatively smooth matrix surface suggests limited plastic deformation. These features are characteristic of interfacial debonding as the dominant damage mechanism.

**Figure 7:**
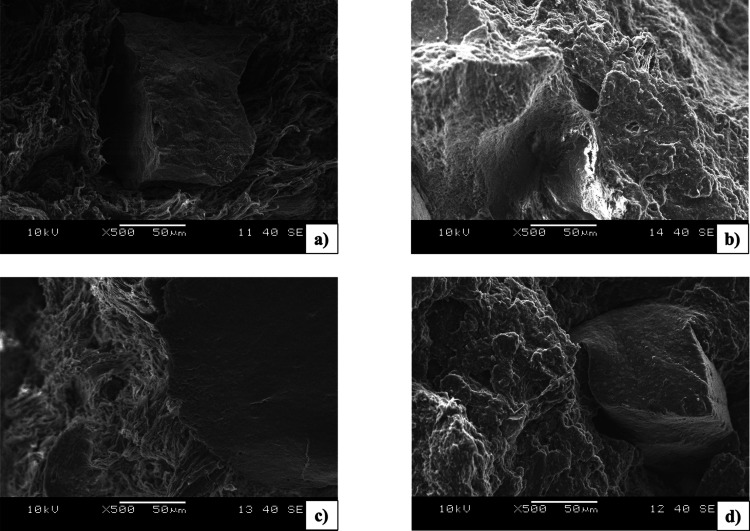
SEM images of the TDVs (a, TDV_ref; b, TDV_TOR2; c, TDV_TOR4; and d, TDV_TOR6; 500× magnification).

With an increase in the TOR content, a gradual improvement in interfacial adhesion can be observed. Although the rubber particles remain distinguishable, the matrix surface surrounding the particles becomes rougher and local matrix deformation is evident. At higher TOR contents, the phase boundary becomes less distinct and the PP matrix shows clear signs of plastic flow and fibrillation around the rubber particles. The reduced incidence of clean particle pullout indicates enhanced interfacial bonding, enabling a more effective stress transfer between the phases. Consequently, the dominant damage mechanism appears to shift from interfacial debonding toward matrix-dominated deformation with a mixed failure mode involving both interfacial debonding and matrix shear deformation at intermediate TOR contents.

### 3.5 Tensile Properties

Tensile testing results (Figure 8 and Table 8) further confirmed the reinforcing and toughening effects of incorporating TOR into the TDVs: both tensile strength and elongation-at-break improved with increasing TOR content. These enhancements indicate improved stress transfer across the rubber–matrix interface and enhanced elasticity, attributable to better interfacial compatibility and possible co-cross-linking (as seen in Table 5). The incorporation of TOR also altered the shape of the curves. While the reference TDV exhibited a steeper initial slope and earlier failure, TOR-modified systems showed reduced initial stiffness combined with significantly extended deformation capability. This behavior indicates improved interfacial stress transfer and a more homogeneous deformation mechanism, leading to enhanced toughness rather than simple reinforcement. These improvements in mechanical properties are consistent with the previously observed increases in cross-link density and enhanced elastic recovery, confirming the structural reinforcement and improved cohesion provided by TOR. The mechanical improvements observed upon TOR incorporation are consistent with previously reported effects of TOR in PP/GTR systems^32^ and compare favorably with other compatibilization strategies reviewed for similar material systems,^24^ supporting the effectiveness of TOR as a reactive compatibilizer for dGTR-based TDVs.

**Figure 8:**
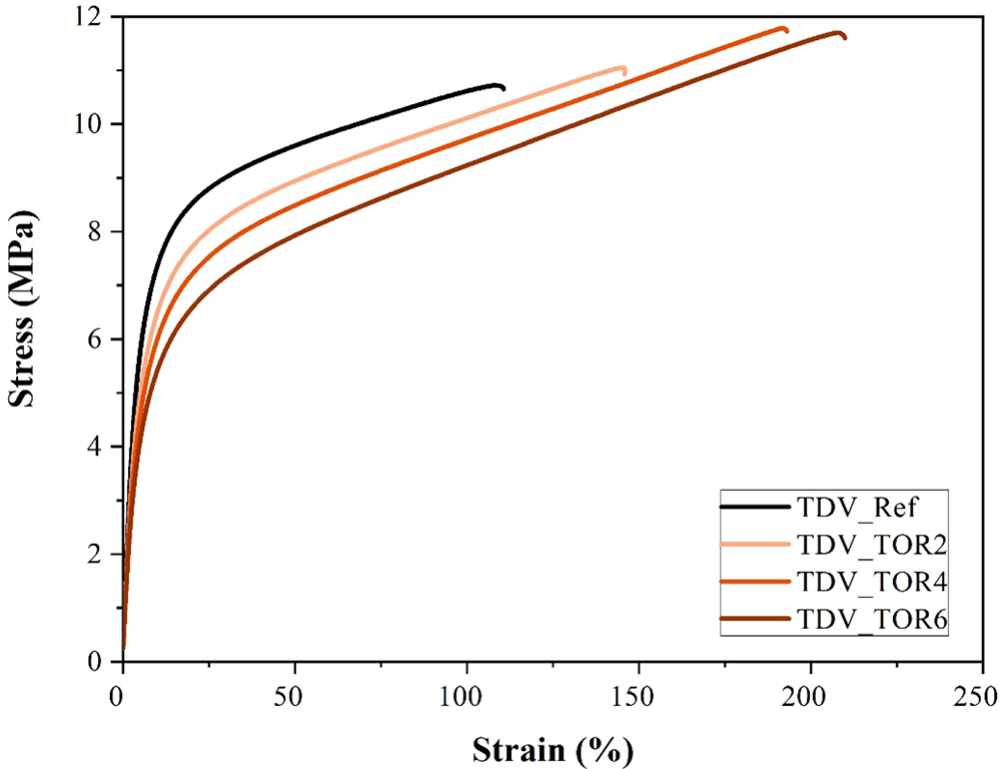
Typical stress–strain curves of the TDVs.

**Table 8:** Mechanical and Impact Properties of the TDVs

	tensile strength (MPa)	strain at break (%)	perforation energy (J/mm)
TDV_Ref	9.5 ± 0.5	125.1 ± 12.3	4.6 ± 1.1
TDV_TOR2	10.8 ± 0.2	150.3 ± 10.1	7.6 ± 0.3
TDV_TOR4	11.5 ± 0.4	192.8 ± 7.6	10.2 ± 0.4
TDV_TOR6	11.5 ± 0.2	210.5 ± 3.7	11.1 ± 0.9

### 3.6 Impact Properties

The incorporation of TOR significantly altered the impact behavior of the TDVs (Figure 9 and Table 8). While the reference material exhibited brittle, catastrophic fracture, TOR-compatibilized systems showed a tougher, more progressive failure with substantially increased deformation at break. This behavior indicates enhanced interfacial adhesion and more efficient stress transfer between the PP matrix and the rubber phase, resulting in an improved energy dissipation during deformation.

**Figure 9:**
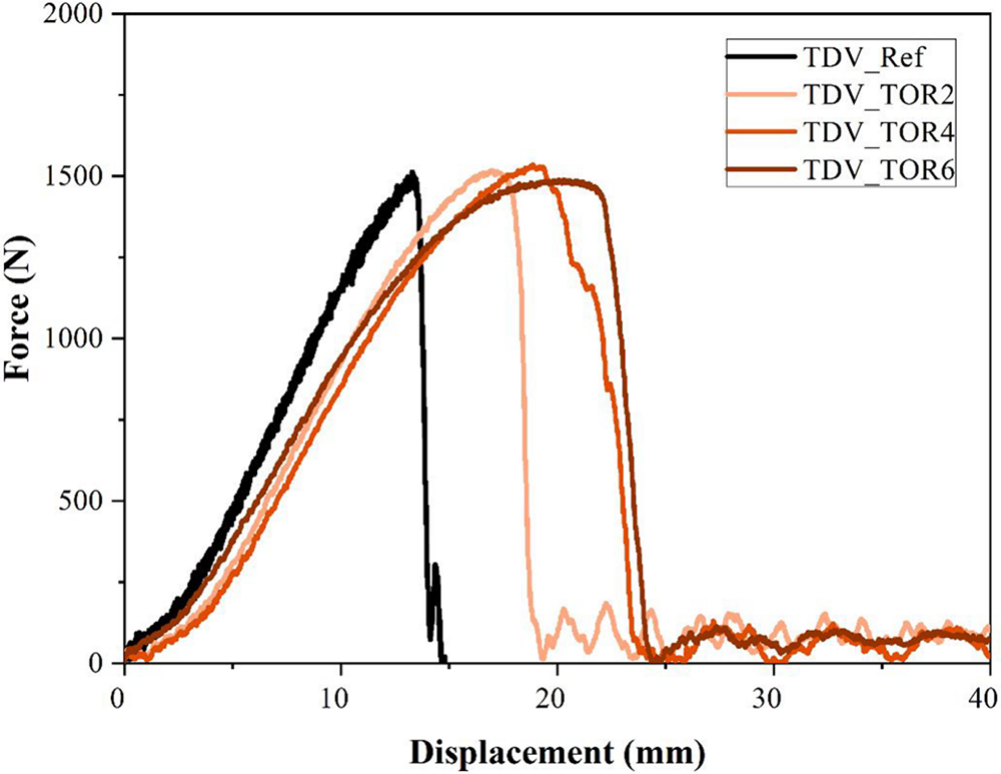
Typical impact curves of the TDVs.

### 3.7 Damage Behavior

The addition of TOR significantly enhanced the deformability and toughness of the TDVs, and AE analysis highlighted a shift in their damage mechanisms compared with the reference material (Figure 10). The reference sample exhibited a rapid, concentrated accumulation of AE events over a relatively narrow strain range, indicating sudden microstructural damage and suggesting a poor interfacial adhesion between the PP and rubber domains. This observation aligns with our previous findings, which demonstrated that the damage behavior of rubber-filled thermoplastic polymers typically occurs in three distinct stages, identified by the number and amplitude of AE signals.^26^ These stages are observable in the present study and indicated by red lines.

**Figure 10:**
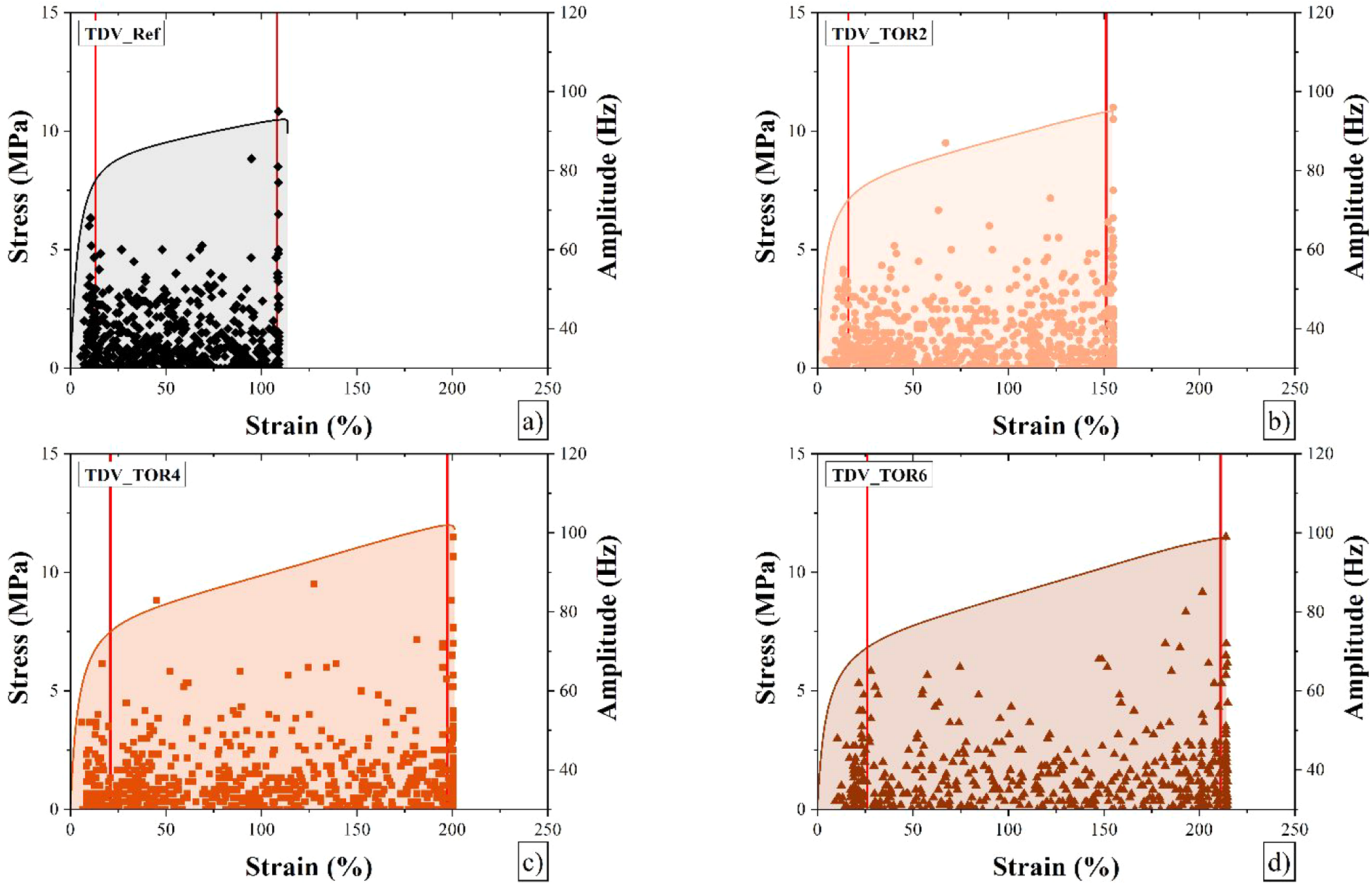
Tensile curves of the TDVs with the corresponding amplitudes (a, TDV_ref; b, TDV_TOR2; c, TDV_TOR4; d, TDV_TOR6).

Incorporating TOR into the TDVs significantly altered the material’s response. The boundary of the first damage stage shifted to higher strains, suggesting enhanced elasticity and a delayed yielding process, which contributes to improved toughness (as seen in the SEM images). AE events in samples containing TOR were more evenly distributed across a broader strain range, reflecting a gradual and controlled progression of damage.

Additionally, increasing the TOR content progressively decreased the AE emission event rate, with the highest TOR concentration showing a significantly lower damage accumulation rate (Figure 11a). This behavior strongly indicates enhanced interfacial adhesion and effective cross-linking between the TOR and rubber phases, thereby stabilizing the fracture process and substantially improving the overall mechanical performance of the TDVs.

**Figure 11:**
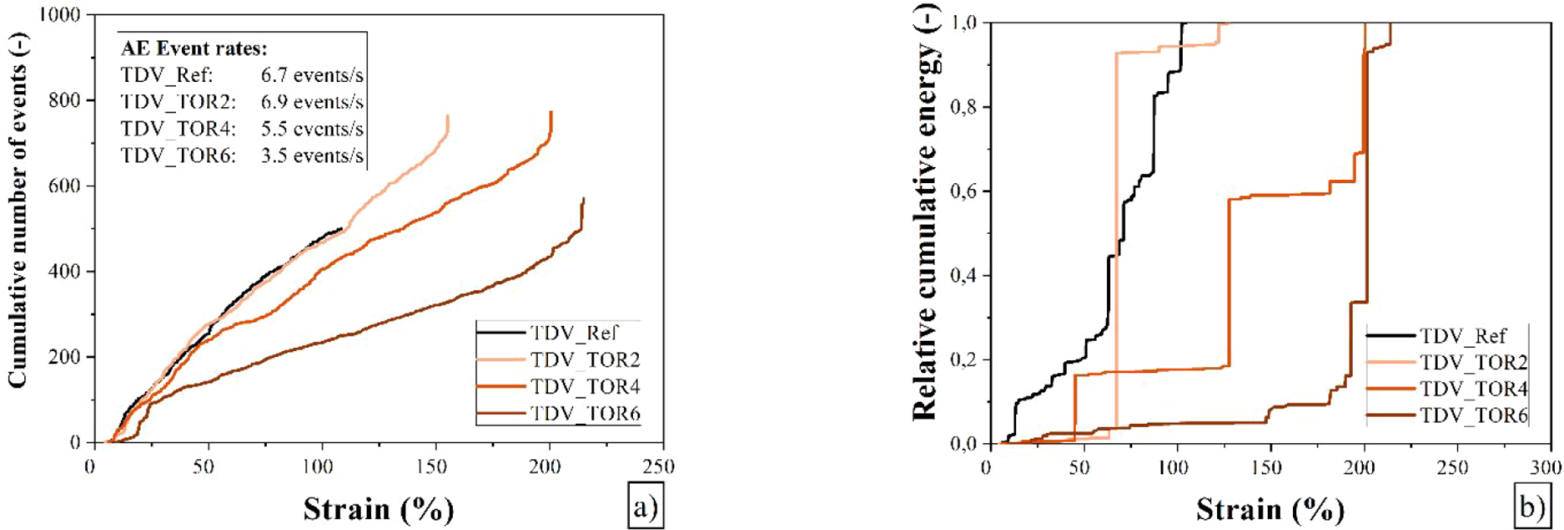
Cumulative number of events (a) and cumulative energy (b) as a function of strain.

The cumulative energy–strain diagram revealed significant differences in the damage mechanisms of TDVs modified with TOR (Figure 11b). The reference sample showed rapid, concentrated accumulation of damage energy. In contrast, increased TOR content led to a slower, more gradual accumulation of damage energy over a much wider strain range. This demonstrates enhanced interfacial adhesion and improved toughness, attributable to effective cross-linking and better interfacial compatibility provided by the TOR. The TDV with the highest TOR content exhibited the most favorable energy-absorbing and stable damage behavior.

The analysis of AE signal properties (Figure 12) aligns with our previous findings.^26^ Incompatible systems (TDV_Ref in this case) generate low-amplitude, short-duration signals and rapid event accumulation, which is typical of more brittle failure. With increased TOR content, the amplitude distribution shifts toward higher values, event durations and rise times elongate, signal strengths improve slightly, and peak frequencies decrease. This indicates an improved compatibility and effective interfacial network formation. The TDV_TOR4 and TDV_TOR6 samples exhibit a smoother and more distributed AE pattern. This indicates that incorporating TOR shifts the damage mechanism toward a tougher, more stable fracture behavior, in line with our earlier findings and those shown in Figure 11.

**Figure 12:**
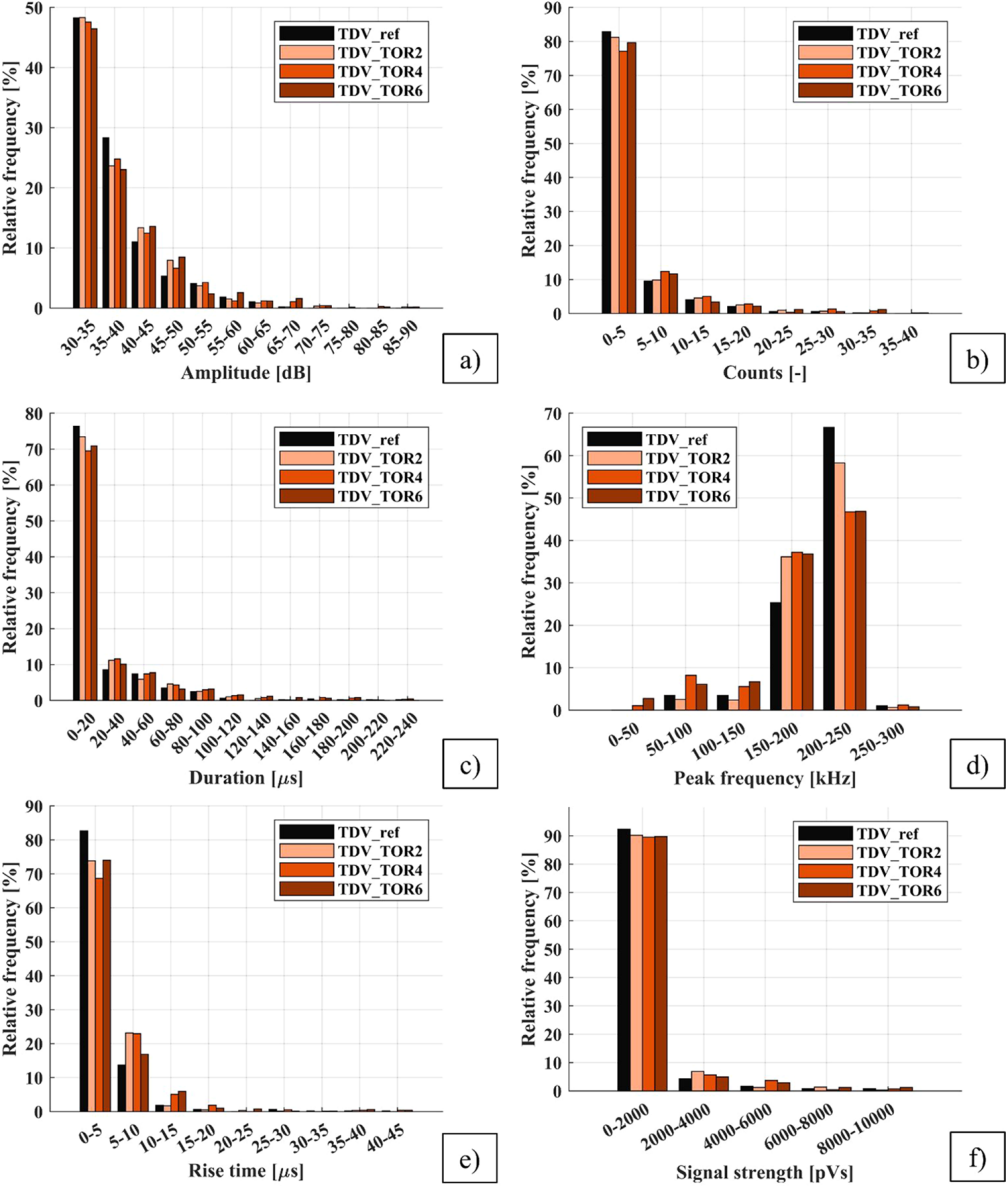
Signal parameters of the specimens (a, amplitude; b, counts; c, duration; d, peak frequency; e, rise time; f, signal strength).

## 4 Conclusions

In this study, we systematically investigated the effect of trans-polyoctenamer (TOR) on the structure, mechanical performance, and damage behavior of PP-based TDVs containing dGTR. Our results demonstrate that TOR plays a multifunctional role in these systems, acting not only as a physical compatibilizer but also as a reactive component actively participating in the formation of the rubber network during dynamic vulcanization.

Anisotherm vulcanization and cross-link density measurements confirmed that TOR is involved in the cross-linking reactions of the rubber phase, leading to an increased cross-link density with increasing TOR content. At the same time, thermal and structural analyses (DSC and XRD) showed that the TOR does not alter the crystalline structure of the PP matrix. These findings indicate that TOR primarily affects the amorphous domains and the interfacial compatibility between the rubber and thermoplastic phases rather than the PP crystalline morphology.

Mechanical testing showed that incorporating TOR results in a pronounced improvement in both strength and deformability. The stress–strain curves showed a systematic transition from a stiffer, more brittle behavior in the reference TDV to a more ductile, tougher response in TOR-modified systems. This change was accompanied by increased elongation-at-break, perforation energy, abrasion resistance, and improved elastic recovery.

AE analysis provided additional insights into the damage mechanisms. The reference TDV exhibited rapid and concentrated damage accumulation, which is characteristic of poor interfacial adhesion and localized failure. In contrast, TOR-modified TDVs showed a delayed onset of damage, a lower event accumulation rate, and a more evenly distributed acoustic response over a wider strain range. These features indicate a more gradual and stable damage process, likely because of improved interfacial stress transfer. Direct characterization of TOR distribution and interfacial and control experiments without cross-linking agents to confirm the role of TOR are identified as a priority for future work.

Overall, incorporating TOR effectively transforms PP/dGTR-based TDVs into tougher materials with enhanced energy absorption and controlled failure behavior. Among the investigated compositions, TOR contents of 4–6 wt % provided the most balanced combination of mechanical performance, elastic recovery, and damage stability. These results demonstrate that TOR is a highly efficient compatibilizer for dGTR-based TDVs and represents a promising tool for the upcycling of end-of-life tires into high-performance, recyclable thermoplastic elastomers. Furthermore, the use of commercially available dGTR and TOR, combined with continuous twin-screw extrusion processing, underscores the industrial scalability and practical relevance of the proposed approach.
